# Cardiac Electrophysiological Alterations in Heart/Muscle-Specific Manganese-Superoxide Dismutase-Deficient Mice: Prevention by a Dietary Antioxidant Polyphenol

**DOI:** 10.1155/2014/704291

**Published:** 2014-03-19

**Authors:** Tadahiro Sunagawa, Takahiko Shimizu, Akio Matsumoto, Motoyuki Tagashira, Tomomasa Kanda, Takuji Shirasawa, Haruaki Nakaya

**Affiliations:** ^1^Research Laboratories for Applied Technology of Food, Asahi Group Holdings, Ltd., Moriya, Ibaraki 302-0106, Japan; ^2^Molecular Gerontology, Tokyo Metropolitan Institute of Gerontology, Itabashi-ku, Tokyo 173-0015, Japan; ^3^Department of Advanced Aging Medicine, Chiba University Graduate School of Medicine, Chuo-ku, Chiba 260-8670, Japan; ^4^Department of Pharmacology, Chiba University Graduate School of Medicine, Chuo-ku, Chiba 260-8670, Japan; ^5^Department of Aging Control Medicine, Juntendo University Graduate School of Medicine, Bunkyo-ku, Tokyo 113-0033, Japan

## Abstract

Cardiac electrophysiological alterations induced by chronic exposure to reactive oxygen species and protective effects of dietary antioxidant have not been thoroughly examined. We recorded surface electrocardiograms (ECG) and evaluated cellular electrophysiological abnormalities in enzymatically-dissociated left ventricular (LV) myocytes in heart/muscle-specific manganese-superoxide dismutase-deficient (H/M-*Sod2* ^−/−^) mice, which exhibit dilated cardiomyopathy due to increased oxidative stress. We also investigated the influences of intake of apple polyphenols (AP) containing mainly procyanidins with potent antioxidant activity. The QRS and QT intervals of ECG recorded in H/M-*Sod2* ^−/−^ mice were prolonged. The effective refractory period in the LV myocardium of H/M-*Sod2* ^−/−^ mice was prolonged, and susceptibility to ventricular tachycardia or fibrillation induced by rapid ventricular pacing was increased. Action potential duration in H/M-*Sod2* ^−/−^ LV myocytes was prolonged, and automaticity was enhanced. The density of the inwardly rectifier K^+^ current (*I*_K1_) was decreased in the LV cells of H/M-*Sod2* ^−/−^ mice. The AP intake partially improved these electrophysiological alterations and extended the lifespan in H/M-*Sod2* ^−/−^ mice. Thus, chronic exposure of the heart to oxidative stress produces a variety of electrophysiological abnormalities, increased susceptibility to ventricular arrhythmias, and action potential changes associated with the reduced density of *I*_K1_. Dietary intake of antioxidant nutrients may prevent oxidative stress-induced electrophysiological disturbances.

## 1. Introduction

Cardiac arrhythmia is a major health problem in the elderly. Although the incidence of ventricular arrhythmias is higher in patients with heart disease, premature ventricular contractions (PVCs) and multiform PVCs are common even in healthy aged people [1–3]. Oxidative stress has been implicated in age-related changes in the electromechanical function of the heart. The generation of reactive oxygen species (ROS) and age-inherent loss in antioxidant capacity may be involved in age-related cardiac dysfunction [4–6]. We previously reported that heart/muscle-specific manganese-superoxide dismutase- (Mn-SOD-) deficient (H/M-*Sod2* ^−/−^) mice exhibited progressive congestive heart failure with the typical pathology of dilated cardiomyopathy [7]. Moreover, biochemical analyses using ROS-reacting reagents revealed that a lack of Mn-SOD induced ROS production in the cardiomyocytes of mutant mice* in vivo* [8]. Therefore, the excess generation of ROS by mitochondria might lead to mechanical dysfunction, that is, congestive heart failure. However, the electrophysiological alterations induced by chronic exposure to ROS have not been evaluated, although several studies have examined the acute effects of ROS on cardiac electrophysiology [9–12]. The present study was undertaken to evaluate the electrophysiological abnormalities induced by chronic exposure to oxidative stress using H/M-*Sod2* ^−/−^ mice.

Antioxidants are currently being widely used as ingredients in dietary supplements in the hope of maintaining health and preventing diseases such as coronary heart disease. Plants synthesize secondary compounds such as polyphenols, which show potent antioxidant activity [13]. It was previously reported that dietary apple polyphenols (AP), which mainly contain procyanidins (PC), suppress ROS production in the cardiomyocytes of H/M-*Sod2* ^−/−^ mice* in vivo* [8]. Another purpose of this study was to examine the effects of chronic intake of AP on electrophysiological alterations in H/M-*Sod2* ^−/−^ mice.

## 2. Materials and Methods

### 2.1. Experimental Animals and Protocol

H/M-*Sod2* ^−/−^ mice were produced by crossbreeding* Sod2* ^flox/flox^ mice with muscle creatine kinase promoter-Cre transgenic mice using* in vitro* fertilization techniques [7, 14]. Control and H/M-*Sod2* ^−/−^ mice were treated with pure water or AP. AP were extracted from apples using the methods reported by Ohnishi-Kameyama et al. [15]. Based on the analysis of the components of AP, PC accounted for 63.8% of AP [16].

Four groups of female mice were used in this study: H/M-*Sod2* ^−/−^, H/M-*Sod2* ^−/−^ treated with AP (H/M-*Sod2* ^−/−^ + AP),* Sod2* ^flox/flox^ (control), and* Sod2* ^flox/flox^ treated with AP (control + AP), respectively. AP were administrated to mice from birth to death. Mice administered the AP treatment were allowed* ad libitum* access to drinking water containing 0.1% AP. All mice were housed in a plastic cage within a pathogen-free barrier facility (23.5 ± 0.5°C) under a 12 h light cycle (08:00–20:00). All procedures for animal care and experimentation conformed to the Guide for the Care and Use of Laboratory Animals published by the United States National Institutes of Health. They were approved by the Animal Care and Use Committee of the Tokyo Metropolitan Institute of Gerontology and the Institutional Animal Care and Use Committee of Chiba University.

### 2.2. Electrophysiological Experiments* In Vivo*

Surface electrocardiograms (ECG) were recorded in 23- to 25-week-old mice anesthetized with urethane (1.5 g/kg, i.p., Sigma Chemical Co., St. Louis, MO) using needle electrodes. ECG signals were amplified with an amplifier (Dual-beam memory oscilloscope VC-10, Nihon Kohden, Tokyo, Japan), and data were collected using Chart v5.5 software (PowerLab System, AD Instruments Pty Ltd, Bella Vista, Australia). ECG parameters including RR, PR, QRS, and QT intervals were measured and the J amplitude was corrected by the R amplitude. The murine ECG has unusual characteristics that make the interpretation difficult [17, 18]. In this study J wave was defined as a secondary slower positive deflection that immediately followed the QRS complex [18]. T wave was defined as a positive or negative intersecting deviation from the isoelectric baseline following the J wave although QT interval might not reflect ventricular repolarization accurately in mice [17]. The most striking change was an altered T wave morphology in the H/M-*Sod2* ^−/−^ mice. Mice whose QT interval was unmeasurable because of overlapping of T wave and P wave were excluded from analysis.

The inducibility of ventricular arrhythmias in* in vivo* hearts was determined in 23- to 25-week-old open-chest mice anesthetized with urethane. They were supported by artificial ventilation using a respirator (SN-480-7, Shinano, Tokyo, Japan). The body temperature was continuously monitored and kept at 37°C using a heating lamp during the experiments. A bipolar stimulating electrode was also attached to the left ventricular surface. The threshold current for electrical pacing was determined by delivering a rectangular pulse of 1 ms duration from a programmable stimulator (Cardiac Stimulator SEC-2102, Nihon Kohden, Tokyo, Japan). The ventricular effective refractory period (ERP), the coupling interval of the longest premature stimulus that failed to be conducted through the entire heart, was measured with 16 basic (S1S1: 75 ms) stimuli and 1 premature (S1S2) stimulus (twice the threshold, 1 ms duration). Starting at 50 ms, the coupling interval of the premature stimulus was reduced in steps of 2 ms until ERP was attained. The inducibility of ventricular arrhythmias was tested by burst pacing at 30 basic (S1S1: 100 ms) stimuli and 5 premature (S1S2: ERP +2 ms) stimuli (2 times threshold, 1 ms duration) using a programmable stimulator (Electronic Stimulator SEN-7203, Nihon Kohden, Tokyo, Japan) and an isolation unit (88-201J, Nihon Kohden, Tokyo, Japan). A series of bursts was repeated 5 times with a recovery period of 30 s between these stimulations. Ventricular tachycardia (VT) was defined as at least ten ectopic ventricular beats. Ventricular fibrillation (VF) was defined as rapid and fragmented ventricular electrograms lasting for at least 1 s.

### 2.3. Patch Clamp Experiments Using Isolated Cardiomyocytes

Left ventricular myocytes were isolated from the hearts of 18- to 22-week-old female mice as previously described [19]. Briefly, hearts were quickly removed from open-chest mice anesthetized with urethane (1.5 g/kg, i.p.) after intravenous administration of 300 U/kg heparin sodium (Novo-Heparin, Mochida Pharmaceutical Co., Tokyo, Japan). After hearts were rapidly washed in cool HEPES-Tyrode solution (in mmol/L: NaCl 143, KCl 5.4, NaH_2_PO_4_·2H_2_O 0.33, MgCl_2_·6H_2_O 0.5, CaCl_2_ 1.8, glucose 5.5, and HEPES 5.0, pH 7.4 with NaOH), they were mounted to a Langendorff perfusion apparatus and perfused* via* the coronary circulation for 15 min with oxygenated Tyrode solution warmed to 37 ± 0.5°C. Hearts were then perfused for 15 min with oxygenated Ca^2+^-free HEPES-Tyrode solution warmed to 37 ± 0.5°C, followed by perfusion for 10–15 min with oxygenated Ca^2+^-free Tyrode solution containing 0.26 mg/mL collagenase (Wako Pure Chemical Industries, Osaka, Japan) warmed to 37 ± 0.5°C. This was followed by perfusion with 50 mL Kraft-Brühe (KB) solution (in mmol/L: KOH 70, L-glutamic acid 50, KCl 40, taurine 20, KH_2_PO_4_ 20, MgCl_2_·6H_2_O 3.0, glucose 10, EGTA 1.0, and HEPES 10, pH 7.4 with KOH). Left ventricular apex tissue was then shaved and minced in KB solution and filtered through a 70 *μ*m nylon mesh (Cell Strainer, BD Biosciences, San Jose, CA). Isolated myocytes were stored in KB solution at 4°C until use. These isolated cells were used within 12 hr after dissociation. Only rod-shaped and quiescent cells were selected for electrophysiological experiments.

Cardiomyocytes isolated from the left ventricular apex were used for current-clamp and voltage-clamp experiments using the whole-cell configuration of patch-clamp techniques, as previously described [19]. Cells were placed in a recording chamber and superfused with extracellular solution at a rate of 1 mL/min. Patch electrodes were fabricated from glass capillaries (7056, Corning Incorporated, Corning, NY) using a vertical puller PC-10 (Narishige, Tokyo, Japan) with a double pull mode and had a resistance of 2–4 MΩ when filled with pipette solution. Electrodes were connected to a Patch/Whole Cell Clamp Amplifier CEZ-2400 (Nihon Kohden, Tokyo, Japan), and data were collected and analyzed using pCLAMP 9.0 software (Axon Instruments, Sunnyvale, CA). Gigaohm seals were developed between the cell membrane and patch pipette and the membrane was ruptured by a more negative pressure. The internal pipette solution for the recordings of action potentials and inward rectifier K^+^ current (*I*_K1_) contained mmol/L: KOH 110, L-aspartate 110, KCl 20, MgCl_2_·6H_2_O 1.0, ATP K_2_·2H_2_O 5.0, phosphocreatine K_2_ 5.0, EGTA 10, HEPES 5.0, and CaCl_2_ 1.42, pH 7.4 with KOH. For action potentials and *I*_K1_ recordings, myocytes were superfused with a standard HEPES-Tyrode solution. Resting membrane potentials and action potentials evoked by 1 ms depolarizing current injections were recorded. The action potential durations at 50% (APD_50_) and 90% repolarization (APD_90_) were measured from cells that did not show spontaneous activity. For the measurement of other outward K^+^ currents, myocytes were superfused with the following external solution. The external solution for outward K^+^ current recordings contained mmol/L: KCl 5.4, KH_2_PO_4_ 0.33, atropine sulphate·H_2_O 0.005, CoCl_2_·6H_2_O 2.0, choline Cl 143, MgCl_2_·6H_2_O 0.5, glucose 5.5, HEPES 5.0, and CaCl_2_ 1.8, pH 7.4 with KOH. The peak outward K^+^ current (*I*_peak_) was designated as the current at 2 ms after 300 ms voltage steps. The steady state K^+^ current (*I*_ss_) was measured at the end of the 300 ms voltage steps. For the measurement of the L-type Ca^2+^ current, myocytes were superfused with an external solution (in mmol/L: NaCl 143, CsCl 5.4, NaH_2_PO_4_ 0.33, MgCl_2_·6H_2_O 0.5, CaCl_2_ 1.8, glucose 5.5, and HEPES 5.0, pH 7.4 with NaOH). The internal pipette solution for L-type Ca^2+^ current recordings contained mmol/L: CsOH·H_2_O 110, L-aspartate 110, CsCl 20, MgCl_2_·6H_2_O 1.0, ATP K_2_·2H_2_O 5.0, EGTA 10, and HEPES 5.0, pH 7.4 with CsOH. The L-type Ca^2+^ current was defined as the peak inward current of 350 ms voltage steps. All electrophysiological experiments were carried out at 36 ± 0.5°C. The capacitance of the membrane was calculated from the steady-state current in response to a ramp pulse (−5 mV/2.5 ms) from 0 mV. All voltage data were corrected according to a liquid junction potential between the pipette solution and external solution.

### 2.4. Connexin 43 Western Blotting and Immunostaining

Protein extracts were prepared from ventricular tissues using extraction buffer composed of 50 mmol/L Tris-HCl (pH 7.4), 150 mmol/L NaCl, 5 mmol/L EDTA, 1% Triton-X 100, and complete protease inhibitor (Roche Diagnostics, Basel, Switzerland). The protein concentration of samples was measured with a DC protein assay kit (Bio-Rad, Hercules, CA). Proteins were separated by SDS-PAGE, transferred to a PVDF membrane and probed with specific antibodies to connexin 43 (Cx43) (1 : 1000; Cell Signaling, Danvers, MA) and actin (1 : 2500; Sigma, St. Louis, MO). Blots were incubated with horseradish peroxidase-conjugated secondary antibodies, and immunoreactive bands were visualized with ECL (GE Healthcare, Buckinghamshire, UK). For histological analysis, heart tissues were immersed in 10% buffered formalin. Fixed tissues were dehydrated, embedded in paraffin, and sectioned into 4 *μ*m slices. Myocardial sections were deparaffinized and rehydrated through a series of xylene and ethanol and treated with the microwave antigen unmasking buffer technique [20]. Slides were immersed with sodium citrate buffer (pH 6.0), boiled in a microwave oven, and heated in an oil bath at 99°C. Slides were washed in water and then rinsed in PBS. After incubation with protein blocking solution, sections were incubated with the primary antibody, Invitrogen Connexin 43 polyclonal antibody (1 : 100; Life Technologies Corporation, Carlsbad, CA), and left overnight at 4°C. The primary antibody was replaced with nonimmune sera for negative controls. Sections were incubated with the secondary antibody, Alexa488 goat anti-rabbit IgG (1 : 100; Life Technologies Corporation, Carlsbad, CA). The intensity was calculated blindly in 14 microscopic sections of the left ventricle from each mouse. The level of fluorescence was normalized to that of the control level.

### 2.5. Statistical Analysis

We used SPSS ver. 11.5 software (IBM SPSS, Armonk, NY). The standard errors and *P* values of the survival data were calculated using the Log-rank test. Differences were considered significant at *P* < 0.05. Statistical comparisons were performed using a one-way analysis of variance (ANOVA). A multiple comparison between groups was performed with Tukey's test as a post hoc analysis. Chi-square analysis was used to compare the incidence of the appearance of automaticity among different groups of myocytes.

## 3. Results

### 3.1. Survival Rate and ECG Changes in H/M-*Sod2* ^−/−^ Mice

**Figure 1:**
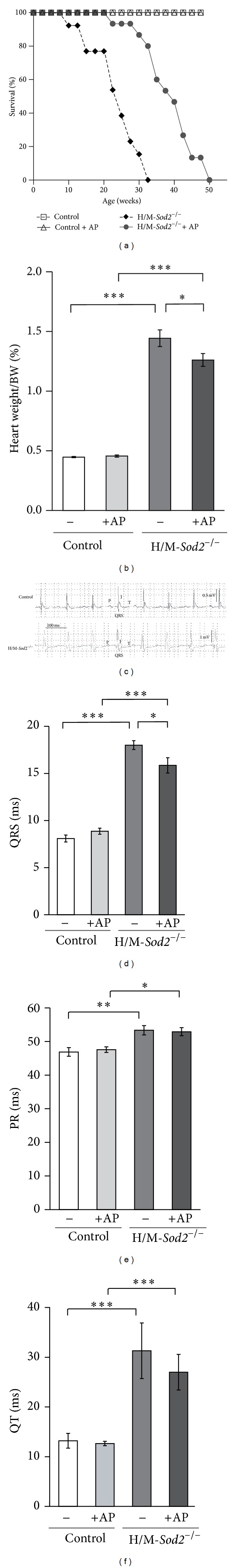
Effects of chronic apple polyphenols (AP) intake on life span, heart weights, and electrocardiograms (ECG) in control and H/M-*Sod2* ^−/−^ mice. (a) Survival curves of 4 experimental groups. Control, *n* = 10, control + AP, *n* = 10, H/M-*Sod2* ^−/−^, *n* = 13, H/M-*Sod2* ^−/−^ + AP, *n* = 15. (b) Heart weight normalized by body weight in 4 experimental groups. Control, *n* = 8, control + AP, *n* = 9, H/M-*Sod2* ^−/−^, *n* = 15, H/M-*Sod2* ^−/−^ + AP, *n* = 15. (c) Representative records of surface ECG (lead II) in control and H/M-*Sod2* ^−/−^ mice. (d) QRS durations, (e) PR intervals, and (f) QT intervals of ECG recorded in 4 experimental groups. ((a)–(e)) Control, *n* = 7, control + AP, *n* = 10, H/M-*Sod2* ^−/−^, *n* = 13, H/M-*Sod2* ^−/−^ + AP, *n* = 12. (f) Control, *n* = 7, control + AP, *n* = 10, H/M-*Sod2* ^−/−^, *n* = 7, H/M-*Sod2* ^−/−^ + AP, *n* = 7. Values are mean ± SEM, ***P < 0.001, **P < 0.01, *P < 0.05.

Heart/muscle-specific manganese-superoxide dismutase-deficient (H/M-*Sod2* ^−/−^) mice showed shorter survival times compared to the control mice and the survival rate was improved by AP intake (Figure 1(a)), which is consistent with our recent report [8]. The mean survival time of H/M-*Sod2* ^−/−^ mice was significantly increased from 22.1 ± 1.9 weeks to 37.9 ± 2.0 weeks by chronic AP intake. Mutant mice showed a greater heart weight/body weight ratio than control mice and it was partially improved by AP intake (Figure 1(b)).

Surface ECG were recorded in 23- to 25-week-old anesthetized mice. A significant prolongation in the QRS, PR, and QT intervals and a flattening of the J wave were observed in H/M-*Sod2* ^−/−^ mice (Figures 1(c), 1(d), 1(e), and 1(f)). The most striking change was an altered T wave morphology in the H/M-*Sod2* ^−/−^ mice. Mice whose QT interval was unmeasurable because of overlapping of T wave and P wave were excluded from analysis. The H/M-*Sod2* ^−/−^ mice whose QT interval was unmeasurable because of overlapping of T wave and following P wave were 5 of 12 mice. AP intake partially reversed these changes in ECG parameters of H/M-*Sod2* ^−/−^ mice, although it did not affect ECG parameters of control mice.

### 3.2. Ventricular ERP and Susceptibility to Ventricular Arrhythmias in H/M-*Sod2* ^−/−^ Mice

**Figure 2:**
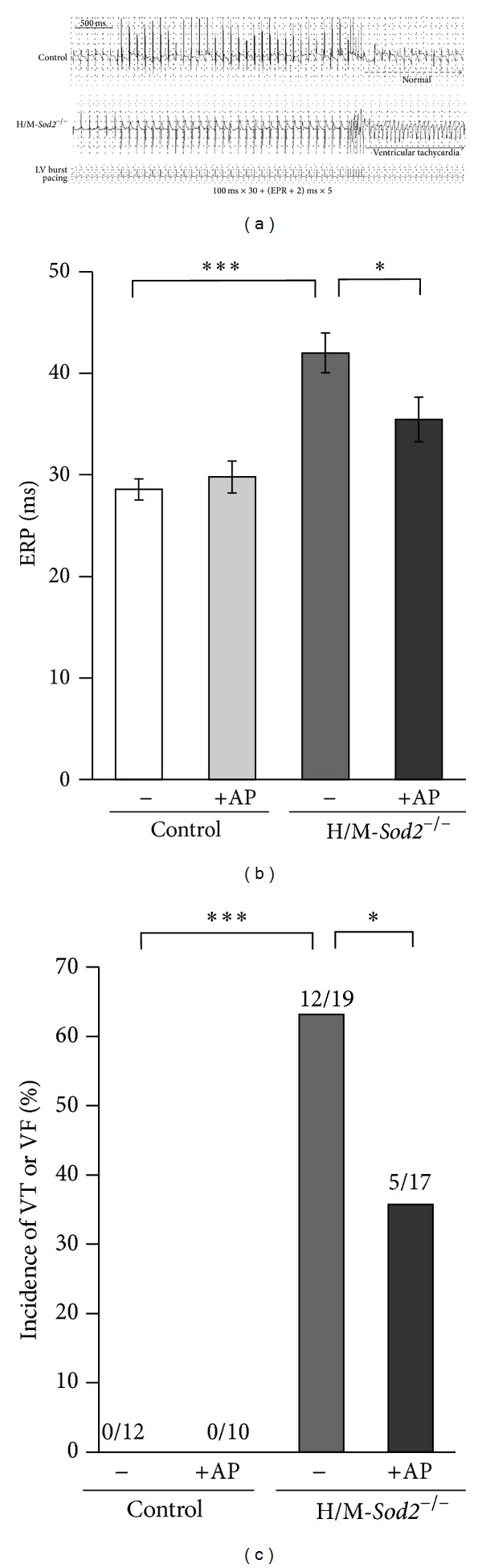
Effects of chronic AP intake on effective refractory period (ERP) and vulnerability to ventricular tachycardia (VT) and ventricular fibrillation (VF) during burst ventricular pacing in control and H/M-*Sod2* ^−/−^ mice. (a) Representative ECG records during burst ventricular pacing observed in a control mouse (upper panel) and H/M-*Sod2* ^−/−^ mouse (lower panel). Note that ventricular tachycardia was induced by burst pacing (30 basic stimuli at a 100 ms interval followed by 5 premature stimuli at an interval of ERP + 2 ms) in the H/M-*Sod2* ^−/−^ mouse but not in the control mouse. (b) Ventricular ERP measured in control and H/M-*Sod2* ^−/−^ mice with and without AP treatment. Control, *n* = 7, control + AP, *n* = 10, H/M-*Sod2* ^−/−^, *n* = 13, H/M-*Sod2* ^−/−^ + AP, *n* = 11. Values are mean ± SEM, ***P < 0.001, *P < 0.05. (c) Incidence of VT or VF induced by burst pacing in control and H/M-*Sod2* ^−/−^ mice with and without AP treatment. ***P < 0.001, *P < 0.05. Note that chronic intake of AP significantly lessened the prolongation in ERP and decreased the incidence of VT or VF induction in H/M-*Sod2* ^−/−^ mice.

In order to investigate whether chronic exposure to oxidative stress affects the repolarization phase of the cardiac action potential, we measured ERP from the left ventricle. Ventricular ERP was significantly prolonged in the hearts of H/M-*Sod2* ^−/−^ mice (42.0 ± 2.0 ms, *n* = 13) relative to that of control mice (28.6 ± 1.0 ms, *n* = 7) (*P* < 0.001) (Figure 2(b)). To determine whether chronic exposure to oxidative stress increases susceptibility to ventricular arrhythmias, we examined the inducibility of VT/VF using a burst ventricular pacing protocol (Figure 2(a)). In control mice treated or untreated with AP, burst pacing failed to induce VT/VF. In contrast, VT/VF could be induced in 12 of 19 H/M-*Sod2* ^−/−^ mice (Figure 2(c)). In AP-treated H/M-*Sod2* ^−/−^ mice, the incidence of VT/VF induction was significantly decreased to 5 of 17 animals (*P* < 0.05).

### 3.3. Action Potential Changes in Ventricular Cells of H/M-*Sod2* ^−/−^ Mice

**Figure 3:**
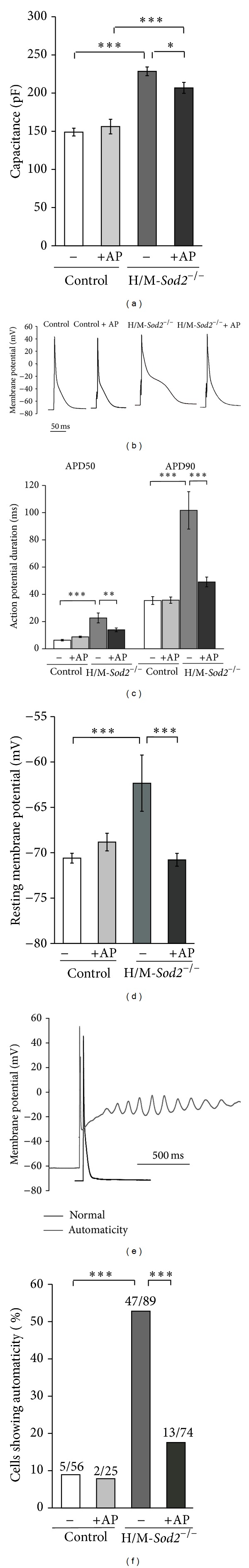
Action potentials recorded from left ventricular cells of control and H/M-*Sod2* ^−/−^ mice and influences of chronic AP intake. (a) The membrane capacitance of ventricular cells isolated from each group of mice. Values are mean ± SEM, control, *n* = 49; control + AP, *n* = 33; H/M-*Sod2* ^−/−^, *n* = 119; and H/M-*Sod2* ^−/−^ + AP, *n* = 72. (b) Representative action potential configurations of the ventricular myocytes in each group. (c) Action potential durations at 50% and 90% repolarization levels (APD_50_ and APD_90_) in the left ventricular myocytes of each group. Control, *n* = 49; control + AP, *n* = 32; H/M-*Sod2* ^−/−^, *n* = 32; and H/M-*Sod2* ^−/−^ + AP, *n* = 41. Differences between groups, ***P < 0.001, **P < 0.01. (d) Resting membrane potentials in the left ventricular myocytes of each group. Data were obtained from ventricular cells not showing automaticity. Control, *n* = 48, control + AP, *n* = 23; H/M-*Sod2* ^−/−^, *n* = 29, H/M-*Sod2* ^−/−^ + AP, *n* = 38. Differences between groups, ***P < 0.001. (e) Representative traces of a normal action potential (control group) and action potential showing abnormal automaticity (H/M-*Sod2* ^−/−^ group). (f) The percentage of left ventricular cells showing automaticity. Differences between groups, ***P < 0.001.

In order to investigate the influence of chronic exposure to oxidative stress on action potential and membrane currents, we conducted whole-cell patch-clamp experiments using enzymatically dissociated left ventricular myocytes. Membrane capacitance (Cm) was used as an indirect index of cell size. Cm values for the left ventricular myocytes of H/M-*Sod2* ^−/−^ mice (223.2 ± 5.6 pF, *n* = 119) were significantly greater than those of control mice (148.9 ± 5.1 pF, *n* = 49) (*P* < 0.001) (Figure 3(a)). Chronic intake of AP partially but significantly reduced the Cm value of H/M-*Sod2* ^−/−^ myocytes to 206.8 ± 7.1 pF (*n* = 72) (*P* < 0.05).

Representative action potentials recorded in the current-clamp mode from 4 groups of mice are shown in Figure 3(b). Both action potential duration at a 50% repolarization level (APD_50_) and that at a 90% repolarization level (APD_90_) in the left ventricular myocytes of H/M-*Sod2* ^−/−^ mice were significantly longer than those of control myocytes (Figure 3(c)). Chronic intake of AP significantly reversed the prolongation of APD_50_ and APD_90_ in the left ventricular myocytes of H/M-*Sod2* ^−/−^ mice, although it did not influence APDs in those of control mice. The resting membrane potential in H/M-*Sod2* ^−/−^ myocytes (−62.3 ± 3.1 mV, *n* = 29) was significantly smaller than that in control myocytes (−70.6 ± 0.5 mV, *n* = 48) (*P* < 0.001). This reduced resting membrane potential was improved by chronic AP intake (Figure 3(d)). Abnormal automaticity such as early afterdepolarizations, probably resulting from incomplete repolarization, was observed in about half of the ventricular cells of H/M-*Sod2* ^−/−^ mice but was rare in control cardiomyocytes (Figures 3(e) and 3(f)). Thus, chronic AP intake significantly inhibited the appearance of abnormal automaticity in H/M-*Sod2* ^−/−^ myocytes.

### 3.4. Changes of Membrane Currents in Ventricular Cells of H/M-*Sod2* ^−/−^ Mice

**Figure 4:**
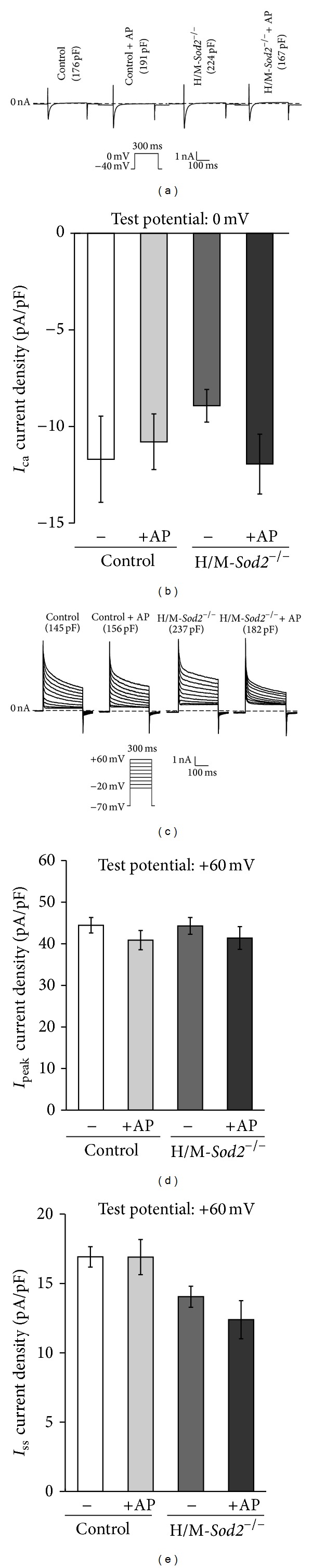
L-type Ca^2+^ and outward K^+^ currents recorded from the ventricular cells of control and H/M-*Sod2* ^−/−^ mice and influences of chronic AP intake. (a) Representative current traces of the L-type Ca^2+^ current recorded from the left ventricular cells of each group. (b) Densities of the L-type Ca^2+^ current of the left ventricular cells of each group. Values are mean ± SEM, control, *n* = 4; control + AP, *n* = 7; H/M-*Sod2* ^−/−^, *n* = 16; and H/M-*Sod2* ^−/−^ + AP, *n* = 9. (c) Representative current traces of the outward K^+^ current recorded from the left ventricular cells of each group. (d) Densities of the peak outward K^+^ current (*I*_peak_) of the left ventricular cells of each group. *I*_peak_ was measured 2 ms after a 300 ms voltage step to +60 mV. (e) Densities of the steady state K^+^ current (*I*_ss_) of the left ventricular cells of each group. *I*_ss_ was measured at the end of a 300 ms voltage step to +60 mV. Control, *n* = 32; control + AP, *n* = 31; H/M-*Sod2* ^−/−^, *n* = 32; and H/M-*Sod2* ^−/−^ + AP, *n* = 32.

**Figure 5:**
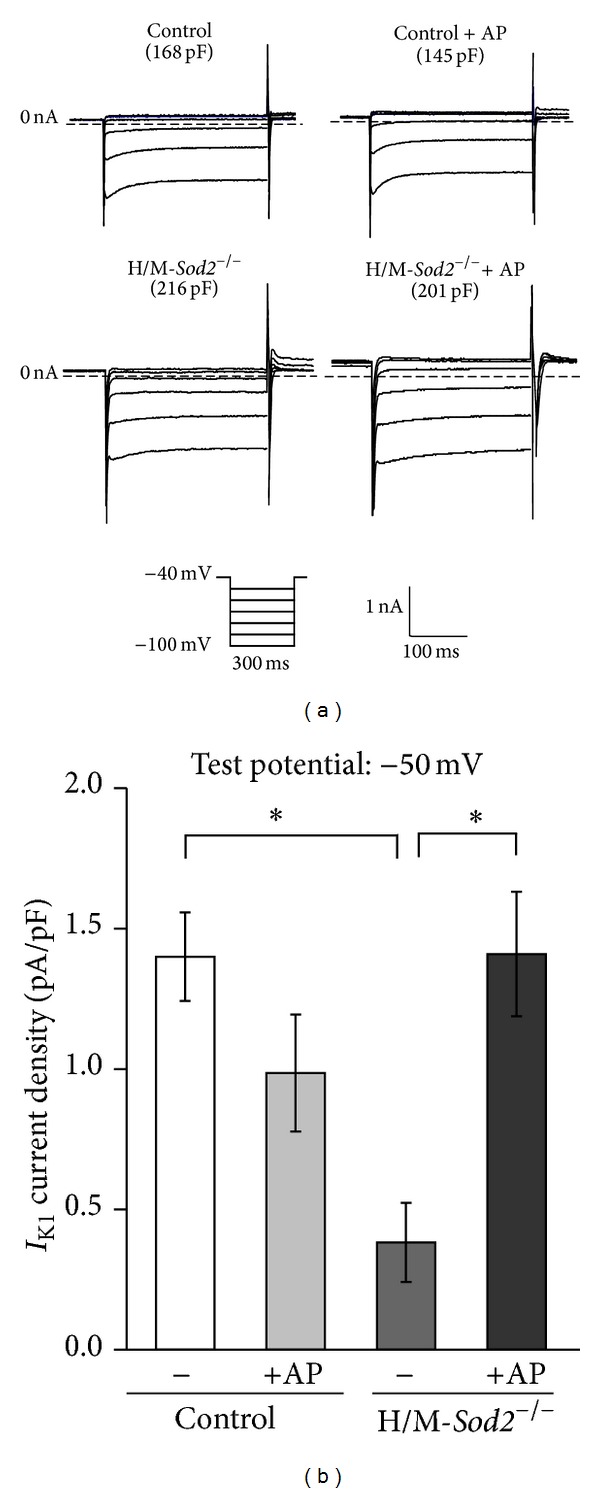
The inward rectifier K^+^ current (*I*_K1_) recorded from the ventricular cells of control and H/M-*Sod2* ^−/−^ mice and influences of chronic AP intake. (a) Representative traces of *I*_K1_ recorded from the left ventricular cells of each group. (b) Densities of *I*_K1_ of the left ventricular cells of each group. The density of *I*_K1_ was measured at the end of a hyperpolarizing pulse to –50 mV from a holding potential of –40 mV. Note that the density of *I*_K1_ in H/M-*Sod2* ^−/−^ ventricular cells was significantly smaller than that in control cells and that the reduction in *I*_K1_ density in H/M-*Sod2* ^−/−^ cells was improved by chronic AP intake. Values are mean ± SEM, control, *n* = 32; control + AP, *n* = 31; H/M-*Sod2* ^−/−^, *n* = 32; and H/M-*Sod2* ^−/−^ + AP, *n* = 32. Differences between groups, *P < 0.05.

Whole-cell membrane currents were recorded from left ventricular myocytes isolated from control and H/M-*Sod2* ^−/−^ mice using patch-clamp techniques. There were no significant differences in the density of the L-type Ca^2+^ currents in LV myocytes among the experimental groups (Figures 4(a) and 4(b)). Outward K^+^ currents were elicited by 300 ms depolarizing pulses from a holding potential of −70 mV in a Na^+^-free and Co^2+^-containing HEPES-Tyrode solution. There were no significant differences in the density of the peak outward current (*I*_peak_) in the ventricular myocytes of the 4 groups (Figures 4(c) and 4(d)). In addition, no significant differences were observed in the density of the steady-state outward current (*I*_ss_) in the myocytes of the 4 groups, although the density of *I*_ss_ in the ventricular cells of H/M-*Sod2* ^−/−^ mice was slightly smaller than that in the ventricular cells of control mice (Figure 4(e)). In contrast, the density of the inwardly rectifier K^+^ current (*I*_K1_) was significantly smaller in the left ventricular myocytes of H/M-*Sod2* ^−/−^ mice than in control mice, as shown in Figure 5. Chronic intake of AP significantly increased the outward component of *I*_K1_ at −50 mV in H/M-*Sod2* ^−/−^ ventricular cells, although it did not increase the density of *I*_K1_ in the ventricular cells of control mice.

### 3.5. Changes of Cx43 Protein in the Ventricle of H/M-*Sod2* ^−/−^

**Figure 6:**
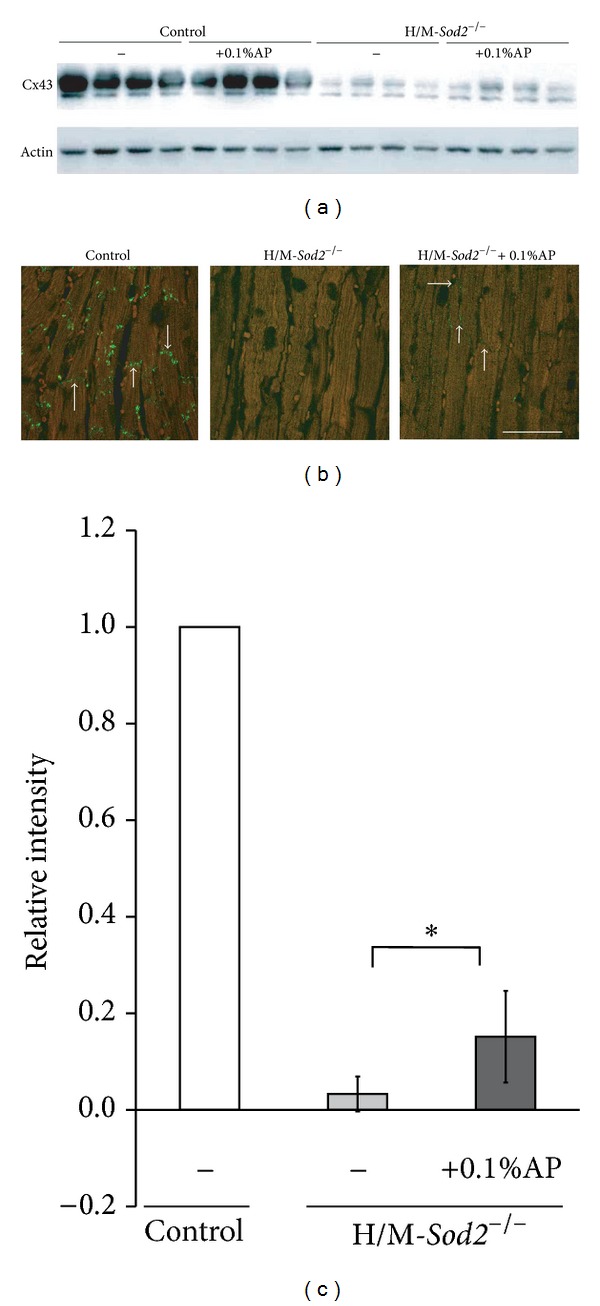
Connexin 43 (Cx43) protein levels in the hearts of control and H/M-*Sod2* ^−/−^ mice. (a) Cx43 levels in the hearts of control and H/M-*Sod2* ^−/−^ mice with and without AP intake. Western blot analysis of Cx43 protein extracted from hearts was carried out using antibodies against Cx43 and actin, *n* = 4. (b) Immunostaining with Cx43-specific antibodies of the left ventricular sections of control and H/M-*Sod2* ^−/−^ mice. Cx43 protein levels in the H/M-*Sod2* ^−/−^ heart relative to the control heart, and chronic 0.1% AP intake partially improved Cx43 expression. (c) Normalized fluorescence intensity for Cx43 immunostaining of the left ventricular sections obtained from H/M-*Sod2* ^−/−^ mice. The fluorescence intensity level was normalized to that determined in the left ventricular sections of control mice. Values are mean ± SEM, *n* = 4, *P < 0.05.

We determined Cx43 protein levels in the hearts of control and H/M-*Sod2* ^−/−^ mice by Western blotting and immunostaining analysis. Western blot analysis of proteins extracted from the ventricles of control and H/M-*Sod2* ^−/−^ mice revealed that the level of Cx43 protein in the H/M-*Sod2* ^−/−^ ventricle was markedly lower than that in the control ventricle (Figure 6(a)). Immunostaining of left ventricular sections with Cx43-specific antibodies showed that the level of Cx43 protein in the H/M-*Sod2* ^−/−^ mouse heart was markedly lower than that in the control mouse heart (Figures 6(b) and 6(c)). Intake of 0.1% AP slightly recovered the level of Cx43 protein in the H/M-*Sod2* ^−/−^ mouse heart (Figures 6(b) and 6(c)).

## 4. Discussion

It is not yet known whether chronic oxidative stress induces cardiac electrophysiological abnormalities including ECG changes. Our study has provided evidence that H/M-*Sod2* ^−/−^ mice exhibit various electrophysiological abnormalities in the heart. The hearts of H/M-*Sod2* ^−/−^ mice showed an increased susceptibility to ventricular arrhythmias during rapid ventricular pacing, concomitantly with ECG changes including prolongation of the QRS and QT intervals and a flattening of the J wave. In the ventricular myocytes of H/M-*Sod2* ^−/−^ mice, APDs were prolonged and the resting membrane potential was decreased, resulting in the appearance of abnormal automaticity. These electrophysiological abnormalities in H/M-*Sod2* ^−/−^ mice were partially reversed by chronic intake of antioxidant AP.

A significant prolongation in the QRS, PR, and QT intervals and a flattening of the J wave were observed in H/M-*Sod2* ^−/−^ mice. The most striking change was an altered T wave morphology in the H/M-*Sod2* ^−/−^ mice. It is well known that there are several differences between humans and rats/mice ECG. In this study J wave was defined as a secondary slower positive deflection immediately following the QRS complex, as proposed by Liu et al. [18]. In a surface ECG of mice, positive J wave was reported to occur during early repolarization [18]. T wave was defined as a positive or negative deviation from the isoelectric baseline, after appearance of J wave. The QT interval may roughly but not precisely reflect ventricular repolarization in mice [17]. Prolonged QT interval might stem from delayed repolarization of ventricular action potentials and increases in duration of QRS complex and PR interval might reflect slowed conduction.

It has been acknowledged that several potassium currents are involved in action potential repolarization in mouse ventricular cells [21, 22]. Voltage-dependent K^+^ channels include Kv4.2, Kv4.3, Kv1.5, and Kv2.1 channels, through which depolarization-induced outward K^+^ currents flow. In the present study, there were no significant differences in the density of the peak outward current (*I*_peak_) or steady-state outward current (*I*_ss_) among ventricular myocytes isolated from the apex of 4 experimental groups of mice. However, the density of the inwardly rectifier K^+^ current (*I*_K1_) in the left ventricular myocytes of H/M-*Sod2* ^−/−^ was significantly smaller than that in control mice. The reduction in *I*_K1_ may be involved in the prolongation of APDs as well as the appearance of abnormal automaticity. Indeed, the resting membrane potential was significantly decreased in H/M-*Sod2* ^−/−^ myocytes. In this context, it is noteworthy that several studies demonstrated a significant suppression of the inwardly rectifier K^+^ channel by acute exposure to oxidative stress [23–25]. The inward rectifier K^+^ channel provides one of the major components of the repolarizing outward current, *I*_K1_, in the cardiac action potential. It was reported that oxidative stress can increase the window current in frog ventricular cells [26]. Therefore, we cannot excluded the possibility that the background sodium current might influence the steady state current around –50 mV although we did not measure the slowly inactivating or late sodium current in the ventricular cells in this study.

In the present study, Cx43 protein was downregulated in the ventricular myocardia of H/M-*Sod2* ^−/−^ mice. A reduction in the gap junctional protein may be in part responsible for depressed ventricular conduction, which was observed as the prolonged QRS complex as well as PR interval in the ECG of H/M-*Sod2* ^−/−^ mice, although we did not measure the density of the fast Na^+^ current (*I*_Na_) in the ventricular cells in this study. Since ROS can reportedly reduce *I*_Na_ [27], the reduction in *I*_Na_ might be also involved in the prolongation of the QRS complex and PR interval in the ECG of H/M-*Sod2* ^−/−^ mice.

The susceptibility to VT/VF during burst ventricular pacing was increased in H/M-*Sod2* ^−/−^ hearts. The dispersion of prolonged APDs as well as depressed conduction in the ventricular myocardium might contribute to the increased susceptibility of H/M-*Sod2* ^−/−^ hearts to VT/VF. The abnormal automaticity observed in ventricular cells isolated from H/M-*Sod2* ^−/−^ hearts might also contribute to the induction of ventricular arrhythmias. Such an increased susceptibility to ventricular arrhythmias can be in part responsible for the shorter life span of H/M-*Sod2* ^−/−^ mice.

Mouse ventricle consists of cardiomyocytes and fibroblasts, and fibroblasts may contribute to fibrosis generation during aging [28]. Oxidative stress and endogenous Mn-SOD regulate collagen synthesis and matrix metalloproteinase activity in cardiac fibroblasts [29, 30]. However, we should point out that the knockout of Mn-SOD was maneuvered only in myocytes in this study.

Chronic intake of antioxidant AP partially prevented electrophysiological alterations such as action potential prolongation, reduced density of *I*_K1_, appearance of abnormal automaticity, and increased susceptibility to VT/VF in H/M-*Sod2* ^−/−^ hearts. AP also lessened the reduction in Cx43 protein in H/M-*Sod2* ^−/−^ hearts. Since ROS levels were lower in the ventricular cells of AP-treated H/M-*Sod2* ^−/−^ mice than in untreated H/M-*Sod2* ^−/−^ mice [8], favorable effects might be ascribed to the antioxidant effects of AP. AP mainly contain procyanidins (PC) with antioxidative activity [31]. Recent reports show that PC are main contributor to the several effects of AP, for example, inhibitory effect on lectin-like oxidized LDL receptor-1, which is a key player in the development of atherosclerosis [32], activating effect on K^+^ channels of aorta endothelial cells [33], and anti-inflammatory effect on intestinal epithelial cells [34]. In this study H/M-*Sod2* ^−/−^ and wild mice were drinking 0.1% (w/v) AP water, and intake of AP was approximately 100~150 mg/kg body weight/day. A previous report showed that apple PC oligomers were detected at a concentration of 11.4 *μ*g/mL in rat plasma after single intake of 1,000 mg AP/kg body weight [35], suggesting that the polyphenol effects would be observed within relevant physiological concentrations. PC are found in suspended red wine and apple juice at high concentrations [36, 37]. From our findings, polyphenols may have value as an ingredient in dietary supplements for the prevention of oxidative stress-induced heart disease.

The incidence of ventricular arrhythmias has increased in response to aging [1–3]. In addition, a recent report indicated that oxidative stress with H_2_O_2_ easily evoked VT/VF in hearts isolated from aged animals [38]. The present study has demonstrated that the chronic oxidative stress may produce various electrophysiological abnormalities in the heart, potentially leading to ventricular arrhythmias. The chronic intake of dietary antioxidants may partly retard the progression of electrophysiological derangements in aged hearts.

## 5. Conclusions

In the H/M-*Sod2* ^−/−^ mice, chronic exposure of the heart to oxidative stress produces a variety of electrophysiological abnormalities including ECG changes, increased susceptibility to ventricular arrhythmias, and action potential changes associated with the reduced density of *I*_K1_. Chronic AP intake partially improved these electrophysiological alterations and extended the lifespan in H/M-*Sod2* ^−/−^ mice. Dietary intake of antioxidant nutrients may prevent oxidative stress-induced electrophysiological disturbances.

## Abbreviations

H/M-*Sod2*^−/−^: Heart/muscle-specific manganese-superoxide dismutase-deficient mice
Mn-SOD: Manganese-superoxide dismutase
ROS: Reactive oxygen species
PC: Procyanidins
AP: Apple polyphenols
APD: Action potential duration
ERP: Effective refractory period
PVC: Premature ventricular contraction
VF: Ventricular fibrillation
VT: Ventricular tachycardia.
